# Supplementary material for: Context-specific interactions in literature-curated protein interaction databases
Source: BMC Genomics. 2018 Oct 19;19:758. doi: 10.1186/s12864-018-5139-2 (PMC6194712; doi:10.1186/s12864-018-5139-2)
Supplement: Supplementary file 1 — Figure S1. Restricting gold standard PPIs to those supported by two or more publications does not eliminate uncorrelated protein pairs, as measured by Pearson correlation R < 0. Each point is one dataset. Horizontal lines show medians. Red: all non-gold standard protein pairs. Black: non-redundant gold standard pairs. “All pairs” and “BioGrid” correspond to Fig. 1. Figure S2. Co-fractionation datasets are more consistent with database PPIs with “co-fractionation-like” evidence codes, e.g. “density sedimentation” and “molecular sieving”. Top: Percent fraction of anti-correlated pairs across co-fractionation datasets, sorted by average percent. Evidence codes are given on the x-axis. Bottom: Average Pearson correlation co-efficient. This analysis is similar to Fig. 2. Each dot represents on co-fractionation dataset. For each evidence code, only datasets with at least 100 database PPIs are shown. All evidence codes with at least one such dataset are shown. Table S1. Some CORUM complexes are predicted by a single high-throughput technique, as measured by average complex coverage. Complex coverage = number of pairwise interactions in a published interactome / total pairwise connections within a complex. Parentheses show number of complexes averaged. CF-specific complexes correspond to numbers 1–80 in Fig. 3d, AP/MS-specific to 81–102, and Y2H-specific to 103–163. To control for expression and bait selection, only complexes that could be be predicted in each interactome are included (see Methods). Figure S3. 60S ribosome co-fractionates via sucrose fractionation (A) but not via heparin dual ion exchange (B). Pearson R. Plots show replicates. Missing (black) are protein pairs where neither protein was detected. (DOCX 561 kb) [file 12864_2018_5139_MOESM1_ESM.docx]

|  | CE-specific complexes | AP/MS-specific complexes | Y2H-specific complexes |
| --- | --- | --- | --- |
| CF interactomes | 0.2863 (474) | 0.0486 (182) | 0.0178 (165) |
| AP/MS interactomes | 0.0075 (85) | 0.2911 (47) | 0.0288 (43) |
| Y2H interactomes | 0.0066 (226) | 0.1348 (81) | 0.2424 (126) |

Table S1. Some CORUM complexes are predicted by a single high-throughput technique, as measured by average complex coverage. Complex coverage = number of pairwise interactions in a published interactome / total pairwise connections within a complex. Parentheses show number of complexes averaged. CF-specific complexes correspond to numbers 1-80 in Figure 3D, AP/MS-specific to 81-102, and Y2H-specific to 103-163. To control for expression and bait selection, only complexes that could be be predicted in each interactome are included (see Methods).


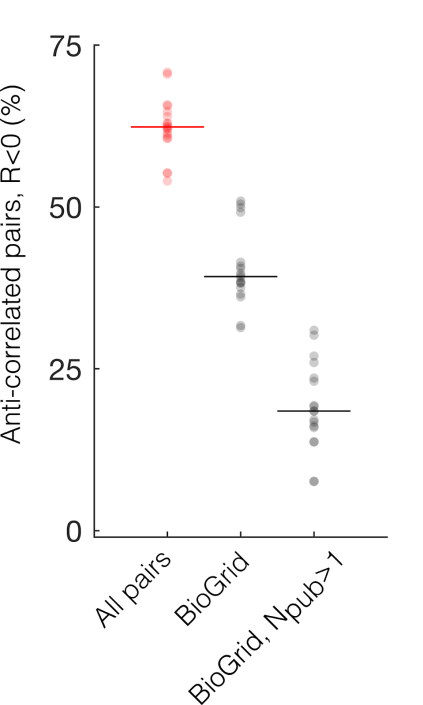


Figure S1 Restricting gold standard PPIs to those supported by two or more publications does not eliminate uncorrelated protein pairs, as measured by Pearson correlation R<0. Each point is one dataset. Horizontal lines show medians. Red: all non-gold standard protein pairs. Black: non-redundant gold standard pairs. “All pairs” and “BioGrid” correspond to Figure 1.


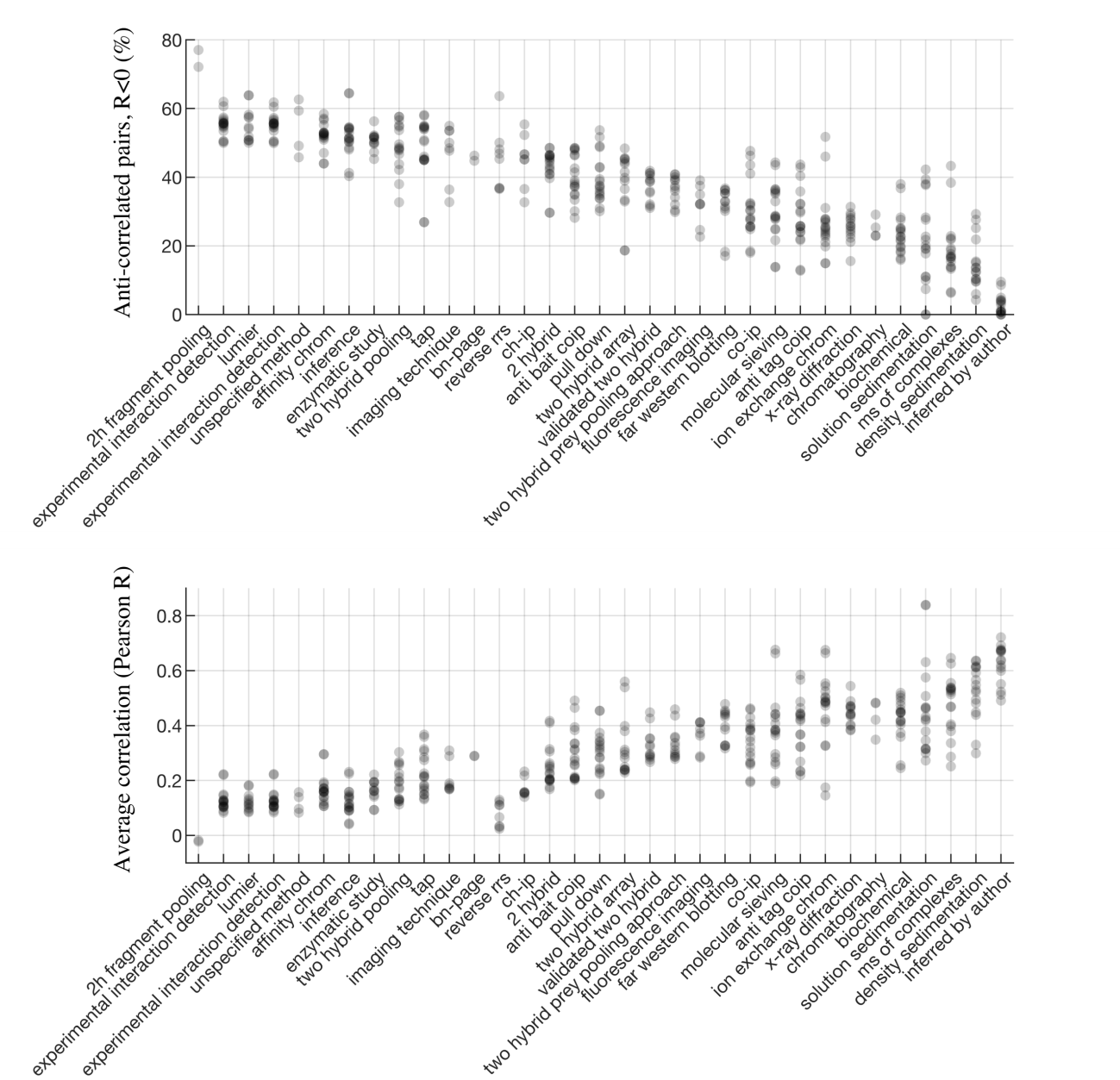


Figure S2. Co-fractionation datasets are more consistent with database PPIs with “co-fractionation-like” evidence codes, e.g. “density sedimentation” and “molecular sieving”. Top: Percent fraction of anti-correlated pairs across co-fractionation datasets, sorted by average percent. Evidence codes are given on the x-axis. Bottom: Average Pearson correlation co-efficient. This analysis is similar to Figure 2. Each dot represents on co-fractionation dataset. For each evidence code, only datasets with at least 100 database PPIs are shown. All evidence codes with at least one such dataset are shown.


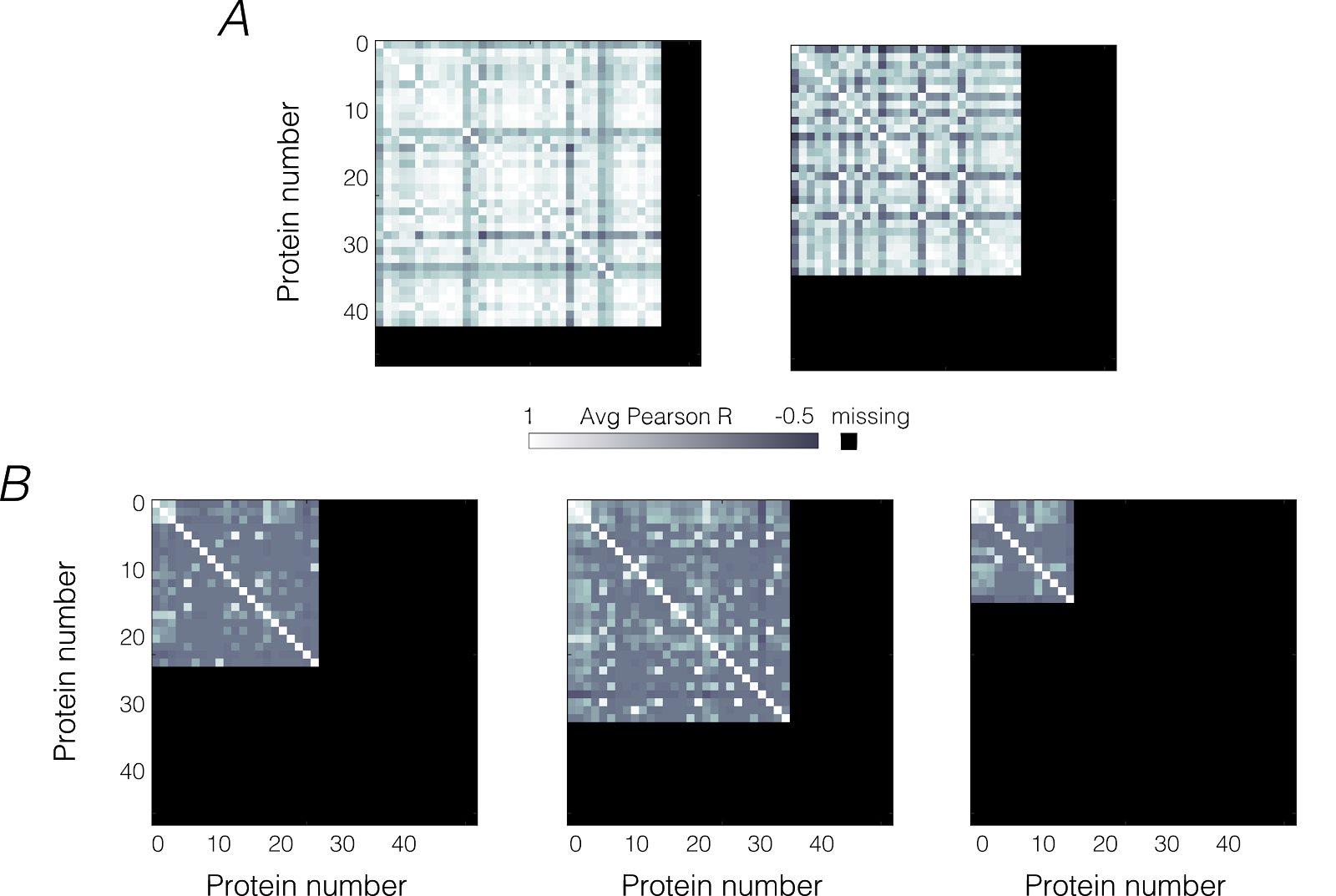


Figure S3. 60S ribosome co-fractionates via sucrose fractionation (A) but not via heparin dual ion exchange (B). Pearson R. Plots show replicates. Missing (black) are protein pairs where neither protein was detected.
